# Supplementary material for: Dietary intake and its associated factors among in-school adolescents in Ghana
Source: PLoS One. 2022 May 12;17(5):e0268319. doi: 10.1371/journal.pone.0268319 (PMC9097987; doi:10.1371/journal.pone.0268319)
Supplement: Attachment — Submitted filename: PONE-D-21-08718_reviewer.pdf [file pone.0268319.s002.pdf]

PLOS ONE

Dietary Intake and Associated Factors among In-school Adolescents in Ghana

--Manuscript Draft--

**Manuscript Number:**	PONE-D-21-08718
**Article Type:**	Research Article
**Full Title:**	Dietary Intake and Associated Factors among In-school Adolescents in Ghana
**Short Title:**	Dietary Practices of In-school Adolescents in Ghana
**Corresponding Author:**	Thomas Hormenu<br>University of Cape Coast, Ghana<br>Cape Coast, Central Region GHANA
**Keywords:**	Diets, Adolescents; Fruits and Vegetable-intake; Socio-demographic Variations; Ghana
**Abstract:**	Background: Early-life nutrition related experiences may fuel the emergence of obesity and type 2 diabetes in adolescence. The adoption of unhealthy dietary practices early in life is an indicator of adverse cardiometabolic health in adulthood. In-school adolescents' dietary practices in Ghana have not been explored extensively despite increasing levels of obesity in adolescents. This study sought to examine dietary practices, socio-demographic disparities and the factors influencing dietary choices among in-school adolescents in Ghana.<br>Methods: A school-based, cross-sectional study was conducted in the Central region of Ghana in 2017. Using multistage sampling procedures, a total of 1,311 in-school adolescents were selected for the study. A modified version of the generic Global School Health Survey questionnaire on dietary practices was adapted and used for data collection. Percentage and frequency counts were used to report on the dietary practices, while Chi-square was used to determine socio-demographic variations in the dietary practices. Binary logistic regression was used to compute the influence of socio-demographic characteristics of respondents on the prevalence of healthy dietary behavior among in-school adolescents.<br>Results: This study revealed the prevalence of healthy dietary practices (49.9%, n = 654) among in-school adolescents in the region. The study also found increased frequency of soft drinks (93%, n = 1220) and toffees/sweets (90%, n = 1183) consumption among in-school adolescents. However, low intake of breakfast (57%, n = 749) was observed among the adolescents. Significant disparities were observed in relation to gender, age, parental communication, academic performance and geographical location in the dietary practices of in-school adolescents. Furthermore, gender (OR = 1.36, P = 0.007), academic performance (OR = 2.19, P = 0.001) and geographical location (OR = 1.79, P = 0.001) were found to be significantly associated with dietary practices among in-school adolescents in the region.<br>Conclusions: Fruits and vegetables consumption was associated with gender, academic performance and geographical location, and these may be a reflection that knowledge on healthy food choices and availability are important factors influencing dietary choices among in-school adolescents. School health policy interventions aimed at improving nutritional status among adolescents and enhanced fruit and vegetable consumption in the country should take into account the potential benefit of increasing availability of fruits and vegetables in schools, while reducing access to sweets and soft drinks in the schools and communities.
**Order of Authors:**	Thomas Hormenu
**Additional Information:**	
**Question**	**Response**
**Financial Disclosure**<br><br>Enter a financial disclosure statement that describes the sources of funding for the work included in this submission. Review	No funder has a role in the study design, data collection, analysis and decision to publish this manuscript

the submission guidelines for detailed requirements. View published research articles from *PLOS ONE* for specific examples.<br><br>This statement is required for submission and **will appear in the published article** if the submission is accepted. Please make sure it is accurate.<br><br>**Unfunded studies**<br>Enter: *The author(s) received no specific funding for this work.*<br><br>**Funded studies**<br>Enter a statement with the following details:<br>• Initials of the authors who received each award<br>• Grant numbers awarded to each author<br>• The full name of each funder<br>• URL of each funder website<br>• Did the sponsors or funders play any role in the study design, data collection and analysis, decision to publish, or preparation of the manuscript?<br>• **NO** - Include this sentence at the end of your statement: *The funders had no role in study design, data collection and analysis, decision to publish, or preparation of the manuscript.*<br>• **YES** - Specify the role(s) played.<br><br>* typeset	
**Competing Interests**<br><br>Use the instructions below to enter a competing interest statement for this submission. On behalf of all authors, disclose any competing interests that could be perceived to bias this work—acknowledging all financial support and any other relevant financial or non-financial competing interests.<br><br>This statement is required for submission and **will appear in the published article** if the submission is accepted. Please make sure it is accurate and that any funding sources listed in your Funding Information later in the submission form are also	No conflict of interest in the conduct of this research and preparation of this manuscript

declared in your Financial Disclosure statement.<br><br>View published research articles from *PLOS ONE* for specific examples.<br><br>**NO authors have competing interests**<br><br>Enter: *The authors have declared that no competing interests exist.*<br><br>**Authors with competing interests**<br><br>Enter competing interest details beginning with this statement:<br><br>*I have read the journal's policy and the authors of this manuscript have the following competing interests: [insert competing interests here]*<br><br>* typeset	
**Ethics Statement**<br><br>Enter an ethics statement for this submission. This statement is required if the study involved:<br><br>• Human participants<br>• Human specimens or tissue<br>• Vertebrate animals or cephalopods<br>• Vertebrate embryos or tissues<br>• Field research<br><br>Write "N/A" if the submission does not require an ethics statement.<br><br>General guidance is provided below. Consult the submission guidelines for detailed instructions. **Make sure that all information entered here is included in the Methods section of the manuscript.**	Field research and University of Cape Coast, Ghana has granted ethical approval for this study [UCCIRB/CES/2016/04]

**Format for specific study types**<br><br>**Human Subject Research (involving human participants and/or tissue)**<br>• Give the name of the institutional review board or ethics committee that approved the study<br>• Include the approval number and/or a statement indicating approval of this research<br>• Indicate the form of consent obtained (written/oral) or the reason that consent was not obtained (e.g. the data were analyzed anonymously)<br><br>**Animal Research (involving vertebrate animals, embryos or tissues)**<br>• Provide the name of the Institutional Animal Care and Use Committee (IACUC) or other relevant ethics board that reviewed the study protocol, and indicate whether they approved this research or granted a formal waiver of ethical approval<br>• Include an approval number if one was obtained<br>• If the study involved *non-human primates*, add *additional details* about animal welfare and steps taken to ameliorate suffering<br>• If anesthesia, euthanasia, or any kind of animal sacrifice is part of the study, include briefly which substances and/or methods were applied<br><br>**Field Research**<br><br>Include the following details if this study involves the collection of plant, animal, or other materials from a natural setting:<br>• Field permit number<br>• Name of the institution or relevant body that granted permission	
**Data Availability**<br><br>Authors are required to make all data underlying the findings described fully available, without restriction, and from the time of publication. PLOS allows rare exceptions to address legal and ethical concerns. See the PLOS Data Policy and FAQ for detailed information.	Yes - all data are fully available without restriction

A Data Availability Statement describing where the data can be found is required at submission. Your answers to this question constitute the Data Availability Statement and **will be published in the article**, if accepted.<br><br>**Important:** Stating 'data available on request from the author' is not sufficient. If your data are only available upon request, select 'No' for the first question and explain your exceptional situation in the text box.<br><br>Do the authors confirm that all data underlying the findings described in their manuscript are fully available without restriction?	
**Describe where the data may be found in full sentences. If you are copying our sample text, replace any instances of XXX with the appropriate details.**<br><br>• If the data are **held or will be held in a public repository**, include URLs, accession numbers or DOIs. If this information will only be available after acceptance, indicate this by ticking the box below. For example: *All XXX files are available from the XXX database (accession number(s) XXX, XXX.).*<br>• If the data are all contained **within the manuscript and/or Supporting Information files**, enter the following: *All relevant data are within the manuscript and its Supporting Information files.*<br>• If neither of these applies but you are able to provide **details of access elsewhere**, with or without limitations, please do so. For example:<br><br>*Data cannot be shared publicly because of [XXX]. Data are available from the XXX Institutional Data Access / Ethics Committee (contact via XXX) for researchers who meet the criteria for access to confidential data.*<br><br>*The data underlying the results presented in the study are available from (include the name of the third party*	All files will be available upon request

and contact information or URL).<br>• This text is appropriate if the data are owned by a third party and authors do not have permission to share the data.<br><br>* typeset	
Additional data availability information:	Tick here if the URLs/accession numbers/DOIs will be available only after acceptance of the manuscript for publication so that we can ensure their inclusion before publication.

Click here to access/download;Manuscript;Manuscript_Diet 2021 Plos One.docx

Dietary Intake and Associated Factors among In-school Adolescents in Ghana

Thomas Hormenu

Department of Health, Physical Education and Recreation, University of Cape Coast, Ghana.

Author's email: thormenu@ucc.edu.gh

ABSTRACT

Background: Early-life nutrition related experiences may fuel the emergence of obesity and type 2 diabetes in adolescence. The adoption of unhealthy dietary practices early in life is an indicator of adverse cardiometabolic health in adulthood. In-school adolescents' dietary practices in Ghana have not been explored extensively despite increasing levels of obesity in adolescents. This study sought to examine dietary practices, socio-demographic disparities and the factors influencing dietary choices among in-school adolescents in Ghana.

Methods: A school-based, cross-sectional study was conducted in the Central region of Ghana in 2017. Using multistage sampling procedures, a total of 1,311 in-school adolescents were selected for the study. A modified version of the generic Global School Health Survey questionnaire on dietary practices was adapted and used for data collection. Percentage and frequency counts were used to report on the dietary practices, while Chi-square was used to determine socio-demographic variations in the dietary practices. Binary logistic regression was used to compute the influence of socio-demographic characteristics of respondents on the prevalence of healthy dietary behavior among in-school adolescents.

Results: This study revealed the prevalence of healthy dietary practices (49.9%, n = 654) among in-school adolescents in the region. The study also found increased frequency of soft drinks (93%, n = 1220) and toffees/sweets (90%, n = 1183) consumption among in-school adolescents. However, low intake of breakfast (57%, n = 749) was observed among the adolescents. Significant disparities were observed in relation to gender, age, parental communication, academic performance and geographical location in the dietary practices of in-school adolescents. Furthermore, gender (OR = 1.36, P = 0.007), academic performance (OR = 2.19, P = 0.001) and geographical location (OR = 1.79, P = 0.001) were found to be significantly associated with dietary practices among in-school adolescents in the region.

Conclusions: Fruits and vegetables consumption was associated with gender, academic performance and geographical location, and these may be a reflection that knowledge on healthy food choices and availability are important factors influencing dietary choices among in-school adolescents. School health policy interventions aimed at improving nutritional status among adolescents and enhanced fruit and vegetable consumption in the country should take into account the potential benefit of increasing availability of fruits and vegetables in schools, while reducing access to sweets and soft drinks in the schools and communities.

Keywords: Diets, Adolescents, Fruits and Vegetable-intake, Socio-demographic Variations, Ghana.

Background

Early-life nutrition experiences may fuel the emergence of obesity and type 2 diabetes in adolescence. Adolescence is a critical period for the formation of lifelong behaviors. It is a period where nutritional needs and explorations increase due to the increased growth rate and changes in body compositions [1]. The dramatic increase in energy and nutrient requirements in this period coincides with other factors that may affect adolescents' food choices and nutrient intake and thus, nutritional status. These factors, including the quest for independence and acceptance by peers, greater time spent at school/playgrounds, and preoccupation with self-image, contribute to erratic and unhealthy eating habits [2]. To help prevent diet-related chronic cardiovascular diseases, studies have posited that healthy eating behaviours, including fruit and vegetable consumption, should be established early in life and maintained during adolescence and adulthood [3].

In a national and population-based survey, it was observed that adolescents often fail to meet dietary recommendations for overall nutritional status and for specific nutrient intakes [4]. According to this study, a large number of adolescents received a higher proportion of energy from fat and/or added sugar and had a lower intake of fruits and vegetable than is recommended. In addition, another study conducted by Buxton [5] on junior high school (JHS) adolescents' dietary practices and food preferences found that the majority (n=515, 62.8%) of the adolescents usually skipped breakfast before going to school and preferred snacks (soft drinks and pastries items). Buxton concluded that the majority of students did not have healthy eating patterns and habits, as they usually skipped breakfast and preferred food products with high sugar and fat content as snacks among other dietary habits.

Recent global figures have shown alarming numbers of obese and overweight children and young people. Contributory factors include lifestyle changes in the consumption of energy dense foods, proliferation of energy dense food near living vicinities of adolescents, and lack of complementary physical activities in the schools and communities. Trends over time regarding dietary practices of adolescents reflect the interplays of environmental and sociocultural trends in food availability, nutritional goals and preferability. A survey has indicated that the total energy intake of sweet snacks and candies has increased, while fruit juice and vegetable consumption has decreased among adolescents due to increased attainability of sweet snacks in their living environment [6]. This trend was postulated by Amos et al. [7] that availability of toffees and soft drinks on the school grounds, as well as in the participants' communities, could contribute to the high intake of soft drinks and toffees in the region.

The growing concern is that dietary habits of most societies have changed considerably during a short period of time due to great economic development, as unhealthy foods such as candies, KFC and other fast food, and soft drinks become more accessible and financially feasible than healthier options, both in schools and communities. This has fueled increased consumption of unhealthy food, and, in turn, increased undesirable health consequences [8, 9].

Although healthy eating is necessary during adolescence, studies have shown that as the individual enters adolescence, their dietary habits often become unhealthier [10, 11]. For young people, healthy eating is particularly important for growth and cognitive development. Eating behaviours adopted during adolescence are likely to be maintained into adulthood, underscoring the importance of encouraging healthy eating as early as possible. Guidelines recommend consumption of at least five portions of fruits and vegetables per day, reduced intake of saturated fat and salt, and increased consumption of complex carbohydrates, as these are essential for adolescent growth and vitality [9].

The global increase in prevalence of obesity in children and adolescents could be attributed to unhealthy dietary practices of adolescents [12]. Obesity in childhood is associated with medical

and psychosocial complications [13] and is likely to track into adulthood [14]. Prevention and intervention programmes for children need to start early, focusing on family and school-based initiatives and other adverse environmental factors that overwhelm behavioural and educational techniques designed to modify diet and increase participation in physical activity [13]. In light of the obesity epidemic, it is perhaps not surprising that dieting is such a prevalent behaviour among children [15], some of whom employ unhealthy dieting strategies to lose weight [16].

Eating habits acquired during adolescence continue into adulthood where the association between diet, disease morbidity, and mortality is well recognized [17]. Healthy eating habits contribute to the physical and emotional health and well-being of adolescents. However, psychosocial changes during adolescence (associated with a need for increased independence), environmental factors (advertising, peer pressure, spending more time away from home), and consuming greater quantities of fast foods and snacks have a combined effect on adolescents' eating patterns and food choices. These factors may put adolescents at increased risk for unhealthy eating habits, resulting in poor nutritional health [18].

In addition, dieting behaviours may have a bearing on the health status of adolescents, as there is widespread concern about excessive dieting among young people. The changes in trends of availability, accessibility and visibility of fast food, candies, cookies and soft drinks in schools and communities have been found to be responsible for the increased prevalence of obesity and cardiovascular disease in the developing world. The extent to which these environmental and sociocultural factors interlock and contribute to the changing trends of dietary choices of adolescents in Ghana is unknown. Therefore, examining dietary practices of in-school adolescents and contributory factors is essential for cost effective, culturally-sensitive policy intervention to decrease unhealthy dietary practices and increase intake and enjoyment of fruits and vegetables in our society.

Materials and methods

A school-based cross-sectional approach was used to select study participants from the accessible population consisting of all in-school adolescents in JHS within the age of 10–15 years. Adolescents at this level of education are between 10–15 years and per this study are found in Forms 1–3 [19]. The study subjects were characterized into three groups; 10–11, 12–13, and 14–15 years. However, all of the students sampled were above 11 years, hence, analysis was done with two groups; 12–13 and 14–15 years [20]. A multistage sampling method was adopted comprising cluster, simple random and convenience procedures to select 1,400 in-school adolescents in JHS in the Central region of Ghana. The sample size was calculated using Cohen 'G' power with an effect size of .40, confidence level of 95% and confidence interval of .05. Yet, 94% (N = 1,311) of the questionnaires were completed, returned and used for the analysis.

Survey Instrument

A modified version of the generic Global School Health Survey [21] questionnaire was adapted to suit the context of this study. The questionnaire consisted of 11 items that solicited responses from the participants. Six items sought socio-demographic information and five items measured respondents' dietary practices, including breakfast intake, fruit and vegetable consumption, frequency of carbonated drink consumption and frequency of sweets/toffees consumption. The internal consistency measure for the instrument yielded a reliability coefficient of Kuder-Richardson [KR20] formula 0.80. This value indicated high reliability and KR20 reliability was suitable for dichotomous variables.

Data collection procedure

Selected schools' headmasters/mistresses were formally notified through the metropolitan, municipal/district education directors. Data collection was done during closing periods of the schools in order not to disrupt contact hours. Participants were asked to answer the questionnaires after research assistants explained the importance of honesty on the part of subjects and enhanced confidentiality on the part the researcher. Participants completed the questionnaires in their classroom and recorded their responses directly by ticking or writing their responses to the questions.

Data analysis

Data was coded and analyzed using the Statistical Package for Social Sciences ([SPSS] version 22.0 for Windows) after pre-screenings were initially piloted to ensure accuracy and appropriateness of the data. In order to make meaning out of the data, participants' responses to how often they eat fruits and vegetables in a week were categorized into healthy and unhealthy dietary practices. Consumption of fruits and vegetable regularly or always was described as healthy dietary practices and unhealthy dietary practices, when consumption was sometimes or never eat fruits and vegetables. Frequency counts and percentages were employed to report on dietary practices among in-school adolescents in the region and Chi-square was used to determine socio-demographic disparities in the dietary practices. Further analysis was done using binary logistic regression to establish the relative influence of the socio-demographic factors on dietary practices among in-school adolescents in the region. The outcome variable was dietary practices (unhealthy vs. healthy) and predictor variables were age, sex, socioeconomic status, parental communication, academic performance, geographical location and religion.

Ethical Approval

The study was approved by the Institutional Review Board, University of Cape Coast. Maintenance of confidentiality and anonymity of the participants was ensured throughout the study. Participation the study was voluntary without any compulsion.

Results and Discussion

Dietary practices were assessed and the responses were aggregated. It was found that 49.9% (n = 654) of in-school adolescents regularly consumed fruits and vegetables (Table 1). Also, high consumption of toffees and soft drinks among in-school adolescents was found, as 90% (n = 1183) of the participants regularly consumed toffees and 93% (n = 1220) regularly consumed soft drinks (Table 1). The result also revealed 57% (n = 749) of participants had never taken breakfast before going to school (Table 1). Early school attendance could be responsible for this outcome.

Table 1: Dietary Practices of In-School Adolescents

**Dietary Practices**	**Regular Intake (Yes)**		**Irregular Intake (No)**	
	**Freq.**	**%**	**Freq.**	**%**
**Fruits and vegetable**	654	49.9	657	50.1
**Carbonated drinks**	1220	93.1	91	6.9
**Sweets/Toffees**	1183	90.2	128	9.8
**Breakfast intake**	562	42.9	749	57.1

N= 1311

Sociodemographic Variations of Dietary Practices

There was found to be a statistically significant difference among students in terms of gender, age, socioeconomic status, parental communication, academic performance, and geographical location. The results showed that 54% (n = 367) of girls engaged in healthy dietary

practices compared to 46% of boys (n = 287) [χ^2 (df = 1) = 7.818, *P* = .005]. This outcome meant that girls were more likely to engage in the healthy dietary practice of eating fruits and vegetables than boys. In addition, 57% (n = 171) of in-school adolescents within the age group of 12-13 years were found to have engaged in healthy dietary practices, compared to those within the age group 14-15+ years [χ^2 (df = 1) = 7.876, *P* = .005]. This outcome reveals that unhealthy dietary practices increase with age. This outcome calls for intensification of school regulation to ban the selling of junk food, toffees and carbonated drinks on school grounds and promote the selling of healthy food, such as fruits and vegetables. In addition, a statistically significant difference was found in terms of parental communication and healthy dietary practices [χ^2 (df = 1) = 5.812, *P* = .016]. It was observed that adolescents with easy parental communication 52% (n = 439) were more likely to consume fruits and vegetables than adolescents with difficult parental communication. Furthermore, students with above average academic performance 59% (n = 240) were found to have engaged in healthy dietary practices than students with average and below average academic performance [χ^2 (df = 2) = 30.682, *P* = .001]. The findings mean that in-school adolescents whose academic performances are above average know the importance of healthy dietary practices. Also, it means that knowledge about the essence of fruits and vegetables increase healthy dietary practices. Finally, differences were found in the geographical location [χ^2 (df = 2) = 26.750, *P* = .001] such that 59% (n = 278) in-school adolescents from middle/central zones consumed fruits and vegetables more than adolescents from the coastal and northern zones. A possible reason could be the higher availability of fruits and vegetables in the middle/central parts of the region.

Nevertheless, there were no statistically significant differences between in-school adolescents in terms of their socioeconomic status and religious affiliation in relation to dietary practices. This result means that to engage in the healthy dietary practice of consuming fruits and vegetables is not dependent on the financial status of the parents or religious affiliation.

Table 2: Sociodemographic Variations in the Dietary Practices

**Factors**	**Dietary Practices**						
	**Unhealthy**		**Healthy**		χ^2	***df***	***P-value***
	f	***%***	***f***	***%***			
**Gender**							
Boys	339	54.2	287	45.8			
Girls	318	46.4	367	53.6	*7.818*	*1*	*.005*
**Age**							
12-13yrs	129	43.0	171	57.0			
14-15+yrs	528	52.2	483	47.8	*7.876*	*1*	*.005*
**SES**							
Low	449	52.0	414	48.0			
High	208	46.4	240	43.6	*3.698*	*1*	*.054*
**PC**							
Difficult	258	54.5	215	44.5			
Easy	399	47.6	439	52.4	*5.812*	*1*	*.016*
**AP**							
Below Ave.	159	63.3	92	36.7			
Average	330	50.6	322	49.4	*30.682*	*2*	*.001*

Above Ave.	168	41.2	240	58.8			
**Religion**							
Christians	585	50.5	574	45.5			
Muslims	72	47.4	80	52.6	*.519*	*1*	*.471*
**Geography Location**							
Southern	253	57.4	188	42.6			
Middle	192	41.0	278	59.0	*26.750*	*2*	*.001*
Northern	212	53.0	188	47.0			

N = 1, 311. AP= Academic Performance and PC= Parental Communication

Socio-Demographic Factors Predict Healthy Dietary Practices among In-school Adolescents.

A binary logistic regression was conducted to find the strength of associations between socio-demographic factors and healthy dietary practices among in-school adolescents in the region. Odds ratios (OR) and confidence intervals (CI) were used to determine the strength of the association. The results in Table 3 show that the whole model significantly predicted healthy dietary practices among in-school adolescents (-2LogL =1747.5, χ^2 = 69.83, *P* = 0.001). Overall, socio-demographic characteristics of in-school adolescents explained 6.9% of the variance in healthy dietary practices in the region as Nagelkerke R^2 indicated a variance of 0.069.

Results showed statistically significant variations in the odds of eating fruits and vegetables within gender, academic performance and geographical location. The result revealed that girls were 1.4 times more likely to eat fruits and vegetables than boys (OR = 1.36, 95% CI = 1.08-1.70, *P* = 0.007). This means that boys are at higher risk of unhealthy dietary practices than girls. Similarly, students with above average academic performance were 2.2 times more likely to engage in healthy dietary practices than those whose performances were below average (OR = 2.19, 95% CI = 1.56-3.08, *P* = 0.001). Also, students with average academic performance were 1.6 times more likely to eat fruits and vegetables than those with below average performance (OR = 1.59, 95% CI = 1.16-2.16, *P* = 0.004). In addition, students in the central part of the region were 1.8 times more likely to engage in healthy dietary practices than those from the southern part (OR = 1.79, 95% CI = 1.37-2.35, *P* = 0.001). Also, adolescents from the northern part of the region were not found to consume fruits and vegetables more than those from the southern part of the region (OR = 1.06, 95% CI = 0.97-1.56, *P* = 0.690). However, no statistically significant variations were found in the odds of eating fruits and vegetables within age (OR = 0.79, 95% CI = 0.60-1.03, *P* = 0.076), religious affiliation (OR = 1.10, 95% CI = 0.77-1.57, *p* = 0.592), parental communication (OR = 1.23, 95% CI = 0.98-1.56, *P* = 0.078) or socioeconomic status (OR = 1.12, 95% CI = 0.88-1.42, *P* = 0.358).

Table 3: Binary Logistic Regression of Socio-Demographic Predictors of Healthy Dietary Practices among In-School Adolescents

Predictors	*N*	*%*	*B*	*Wald*	*OR*	*95% CI*	*Sig.*
**Age**							
12-13years (ref)	171	26.1					
14-15years	483	73.9	0.243	3.153	.785	.60-1.03	0.076
**Gender**							
Boys (ref)	287	43.8					

Girls	367	56.2	0.309	7.30	1.36	1.1-1.70	0.007
**Religious Affiliation**							
Christian (ref)	574	87.8					
Muslims	80	12.2	0.960	0.288	1.10	0.77-1.56	0.592
**Parental Communication**							
Difficult (ref)	215	32.9					
Easy	439	67.1	0.210	3.09	1.23	0.97-1.56	0.078
**Socioeconomic Status**							
Low (ref)	414	63.3					
High	240	36.7	0.112	.843	1.11	0.88-1.42	0.358
**Academic Performance**							
Below average (ref)	92	14.1		20.52			0.001
Average	322	49.2	0.460	8.502	1.59	1.2-2.16	0.004
Above average	240	36.7	0.784	20.42	2.19	1.6-3.08	0.001
**Geographical Location**							
Southern (ref)	188	28.7		21.64			0.001
Central	278	42.5	0.583	17.65	1.79		0.001
Northern	188	28.7	0.057	.159	1.06		0.690
Constant			0.864	16.43	.422		0.001

Source: Field Survey 2017

Discussion

Close to half of the participants practiced healthy consumption of fruits and vegetables. This finding is consistent with previous findings that healthy dietary practices regarding fruit and vegetable consumption have hovered around 40-50% among adolescents globally [22, 23, 24]. The findings also mean that half of the participants did not consume fruits and vegetables regularly but quite a substantial number consumed carbonated drinks and toffees regularly. This means that most adolescents are not consuming the recommended servings of fruits and vegetables and could be deprived of the protective benefits of fiber in their diets, making them at higher risk for obesity and cardiovascular diseases [8]. The high level of soda and sweets consumption could subsequently lead to increase prevalence of obesity and cardiovascular diseases among adolescents.

A low intake of breakfast (n=562, 43%) was also found among the students. A possible reason for low breakfast consumption among students in the region might be due to the early school start time and the distance students commute to arrive at school. This finding is inconsistent with the Intiful and Lartey [25] research reports, where they found high breakfast consumption among SHS adolescents. A possible reason for this difference could be that the students sampled in the previous study were at boarding school where breakfast is compulsory. Another reason for low breakfast consumption in the region could be due to poverty [25]. Breakfast is essential for students' concentration in class. Students who skip breakfast lack essential nutrients for the brain, as a good breakfast gives students physical energy and boosts their mental capacity. With this, it is essential for parents to ensure that their children eat a healthy breakfast every day to improve their child's health status, concentration in class and academic performances.

Socio-demographic variations in dietary intake

This study observed disparities in terms of gender, age, socioeconomic status, parental communication, academic performance, and geographical location in relation to healthy dietary practices. These disparities are critical in terms proffering appropriate interventions to enhance healthy dietary practices and reduce prevalence of adolescent obesity. The specific finding that the girls had healthier dietary practices than the boys corroborates with Ranjit et al. [26] and Nilsen et al. [27] that the healthy dietary practice of fruit and vegetable intake is often higher among girls compared to boys. This observation prompts the need for increased physical activity in school among boys to increase glucose metabolism and reduce the effect of unhealthy dietary practices on their body weight.

The finding that the younger age group had healthier dietary practices than the older age group supports the findings of Ranjit et al. [26] and Pearson et al. [28] that younger adolescents engaged in healthier dietary practices than older ones. Harnack et al. [29] explained that soft drink and fast food consumption increased with age in adolescents in the United States. Similarly, Bere et al. [30] posited that fast food consumption and the frequency of visiting fast food restaurants was higher among students in upper classes (ages 15-18) than students in lower classes (ages 12-14).

In terms of academic performance, the finding that students with above average academic performance had healthier dietary practices than those with average or below average performance is consistent with Falkner et al. [31] who found that unhealthy dietary behaviors are always found among students who have had to repeat their grade.

The adolescents in the Central Region did not differ in terms of their socioeconomic status and dietary practices. This finding contrasts with the finding of Henningsen [32] that adolescents with low socio-economic position have a higher consumption of soft drinks and fast food than those with high socio-economic position. Also in contrast with this study, but similar to Henningsen, Fahlman et al. (2010) also found that those with high socioeconomic position engaged in healthier dietary practices.

Predictors of Dietary Intake

Gender was a significant predictor of healthy dietary practices among in-school adolescents in the Central region with boys at more risk of engaging in unhealthy dietary practices. This outcome corroborates with previous research findings [34]. These studies reported that girls were more likely to engage in the healthy dietary practice of eating fruits and vegetables than boys. The plausible explanation to these similarities could be that girls prefer to eat at home compared to boys [34]. Although Layade and Adeoye [35] also found gender as a predictor of healthy dietary practices, they posited that males were more likely to consume fruits and vegetables than females. The possible reason for this difference in the finding could be due to the fact that the current study sampled JHS students while the previous study sampled tertiary students. The outcome also means that boys would lack essential nutrients from fruits and vegetables and suffer related consequences, resulting in the need to promote fruit and vegetable consumption among boys.

Furthermore, academic performance of in-school adolescents also predicted healthy dietary practices in the region, as adolescents with above average academic performance were more likely to eat fruits and vegetables than those with average or below average academic performance. This finding is consistent with previous research findings of Doku et al. [36] that academic performance of adolescents was a significant predictor of healthy dietary practices among adolescents, with students having above average academic performance being more likely to consume fruits and vegetables. The confirmatory reason for this outcome could be that students with high academic

performance are aware of the importance of fruits and vegetables in their diet, and this could be responsible for high consumption of fruits and vegetables among this group [31]. Another plausible reason for the similarities in the findings could be that students with high academic performance eat fruits and vegetables because their parents can afford to purchase them. Therefore, there is a need for education on the importance of fruits and vegetables among students with average and below average academic performance.

Furthermore, this study also found that in-school adolescents in the southern part of the region were at higher risk of not eating fruits and vegetables than adolescents from the central part of the region. This finding is in support of the previous research findings by Layade and Adeoye [35] and Othman et al. [37] on fruit and vegetable consumption by students in Nigeria, where they described geographical location and availability of fruits and vegetables in the locality as a predictor of fruit and vegetable consumption among adolescents. This explains that availability and access to fruits and vegetables in the locality determine students' consumption. Similarly, the study affirms the research report of Currie et al. [38] for HBSC, that the engagement in health behaviours patterned geographical differences, hence the explanation that certain health behaviours depicted certain patterns across geographical locations. This meant that health outcomes showed the pattern of life, culture, traditions and weather conditions of the geographical zones of people. Also, the outcome could be due to no or limited availability of fruits and vegetables on school campuses in the southern part of the region, contributing to inhibited intake, hence, there is a need to encourage the consumption of fruits and vegetables in the school. Nutritional needs during adolescence are increased due to the increased growth rate and changes in body composition associated with puberty [2]. However, poor dietary practices among school adolescents could contribute to obesity. Fruit and vegetable consumption should be promoted in our schools, especially among boys and adolescents living in the northern and southern parts of the region.

Limitations

The cross-sectional approach adopted could limit the generalizability of the predictors of fruits and vegetable consumption among in-school adolescents. Although the utilization of self-recall on the dietary practices of adolescents is useful in determining intake over a long period of time, it lacks detailed information on specific foods and beverages and memory biases and other social desirability issues inherent in the study may lead to over or under reporting of dietary practices.

Conclusion

The findings from this school-based survey have revealed that there is low fruit and vegetable intake and high consumption of sweets and carbonated drinks in Ghana among adolescents. These have the propensity of increasing adolescent obesity and cardiovascular diseases. There is a need to increase availability of fruits and vegetables in schools to compliment the low intake that may occur at home. Also, nutritional education may be a suitable focus for the improvement of dietary behaviors among adolescents in the country. Fruit and vegetable consumption was associated with gender, academic performance and geographical location, and these may be a reflection that knowledge and availability are important factors influencing dietary practices and food choices. School health policy interventions aimed at improving nutritional status among adolescents and enhanced fruit and vegetable consumption in the country should take into account the potential

benefit of increasing availability of fruits and vegetables in schools, while reducing access to sweets and soft drinks in schools and communities.

Conflict of Interest

The author does not have any conflict of interest.

References

1. Jenkins S, Horner SD. Barriers that influence eating behaviours in adolescents. J Pediatr Nurs. 2005; 20, 258.
2. Spear BA. Adolescent growth and development. J Am Diet Assoc. 2002; 102, S23.
3. McNaughton SA, Ball K, Mishra GD, Crawford DA. Dietary patterns of adolescents and risk of obesity and hypertension. J Nutr. 2008; 138, 36-40.
4. Kann L, Kinchen S, Shanklin SL. Youth risk behavior surveillance-United States, 2013. MMWR Surveill Summ. 2014; 63(4):1-8.
5. Buxton CNA. Ghanaian Junior High School adolescents' dietary practices and food preferences: Implications for public health concern. J Nutr Food Science. 2014; 4, 297. doi: 10.4172/2155-9600.1000297.
6. Slining MM, Mathias KC, Popkin BM. Trends in food and beverage sources among US children and adolescents: 1989-2010. J Acad Nutr Diet. 2013; 113, 1683.
7. Amos PM, Intiful FD, Boateng L. Factors that were found to influence Ghanaian adolescents' eating habits. SAGE Open. 2012; 1-6. DOI: 10.1177/2158244012468140.
8. Hagan JE, Nsiah-Asamoah C, Hormenu T, Pollmann D, Schack T. Managing overweight and obesity in Ghana from a cultural lens: The complementary role of behaviour modification. Journal of Preventive Medicine and Care. 2018; 2(2): 1-18. doi: 10.14302/issn.2474-3585.jpmc-18-2059.
9. Kabwama, SN, Bahendeka SK, Wesonga R, Mutungi G, Guwatudde D. Low consumption of fruits and vegetables among adults in Uganda: findings from a countrywide cross-sectional survey. Archives of Public Health. 2019; 77, 4.
10. Rasmussen M. Determinants of fruit and vegetable consumption among children and adolescents: a review of the literature. Part 1: Quantitative studies. International Journal of Behavioral Nutrition and Physical Activity. 2006; 3(22):109-115.
11. Vereecken CA, Inchley J, Subramanian SV, Hublet A, Maes L. The relative influence of individual and contextual socio-economic status on consumption of fruit and soft drinks among adolescents in Europe. European Journal of Public Health. 2005; 15(3): 224-232.
12. Ogden C, Flegal K, Carroll M. Prevalence and trends in overweight among US children and adolescents, 1999–2000. JAMA. 2002; 288, 1728-1732.
13. Ebbeling CB, Pawlak DB, Ludwig DS. Childhood obesity: public-health crisis, common sense cure. Lancet. 2002; 360, 473-482.
14. Dietz, WH. Overweight in childhood and adolescence. N Engl J Med. 2004; 350, 855-857.
15. Crow S, Eisenberg ME, Story M. Psychosocial and behavioural correlates of dieting among overweight and non-overweight adolescents. Journal of Adolescent Health. 2006; 38, 569-574.
16. Eaton DK, Kann L, Kinchen S. Youth risk behavior surveillance – United States, 2007. Surveillance summaries. MMWR. 2008; 57, 1-131.
17. Vereecken CA, Inchley J, Subramanian SV, Hublet A, Maes L. The relative influence of individual and contextual socio-economic status on consumption of fruit and soft drinks among adolescents in Europe. European Journal of Public Health. 2005; 15(3): 224-232.

18. Neumark-Sztainer D, Wall M, Story M, Fulkerson JA. Are family meal patterns associated with disordered eating behaviours among adolescents? Journal of Adolescent Health. 2003; 35, 350-359.
19. Hormenu T, Hagan Jnr JE, Schack T. Predictors of alcohol consumption among in-school adolescents in the Central Region of Ghana: A baseline information for developing cognitive-behavioural interventions. PLoS ONE. 2018; 13 (11): e0207093.
20. Hormenu T, Hagan Jnr JE, Schack T, Dietmar P. Psychosocial determinants of marijuana utilization among selected junior high school students in the central region of Ghana. Journal of Preventive Medicine and Care. 2018a; 2(2): 1-18
21. GSHS. Global school-based student health Survey: Ghana junior high 2012 fact sheet. Accra: CDC. 2012.
22. HBSC. Report on the health behaviour in school-aged children (HBSC) study. Copenhagen, 2 May 2012: WHO Regional Office for Europe. 2012.
23. Kotecha PV, Patel SV, Baxi RK, Mazumdar VS, Shobha M, Mehta KG, Mansi D, Ekta, M. Dietary pattern of schoolgoing adolescents in urban Baroda, India. J Health Population Nutrition. 2013; 31(4): 490-496.
24. WHO. Addressing the socioeconomic determinants healthy eating habits and physical activity levels among adolescents. Geneva: The Regional Office for Europe of the World Health Organization. 2006.
25. Intiful FD, Lartey A. Breakfast habits among school children in selected communities in the Eastern Region of Ghana. Ghana Medical Journal. 2014; 48(2): 71-77.
26. Ranjit, N, Evans MH, Byrd-Williams C, Evans AE, Hoelscher DM. Dietary and activity correlates of sugar-sweetened beverage consumption among adolescents. Pediatrics. 2010; 126(4): 754-761. doi: 10.1542/peds.2010-1229.
27. Nilsen SM, Krokstad S, Holmen TL, Westin S. Adolescents' health-related dietary patterns by parental socio-economic position, The Nord-Trøndelag Health Study (HUNT). European Journal of Public Health. 2009; 20(3): 299-305.
28. Pearson N, Atkin AJ, Biddle SJ, Gorely T, Edwardson C. Patterns of adolescent physical activity and dietary behaviors. International Journal of Behavioural Nutrition and Physical Activity. 2009; 6(45): 458-463.
29. Harnack L, Stang J, Story M. Soft drink consumption among US children and adolescents: nutritional consequences. Journal of American Dietetic Association. 1999; 99, 436-441.
30. Bere E, Glomnes ES, te Velde SJ, Klepp KI. Determinants of adolescents' soft drink consumption. Public Health Nutrition. 2006; 11(1): 49-56.
31. Falkner NH, Neumark-Sztainer D, Story M, Jeffrey RW, Beuhring T, Resnick MD. Social, educational, and psychological correlates of weight status in adolescents. Obesity Research. 2001; 9, 32-42.
32. Henningsen, M. Dietary habits in adolescence related to sociodemographic factors, physical activity and self-esteem. Thesis submitted for the Master Degree in Health Science, Department of Social Work and Health Science, Faculty of Social Science and Technology Management, Norwegian University of Science and Technology, Trondheim. 2011.
33. Fahlman MM, McCaughtry N, Martin J, Shen B. Racial and socioeconomic disparities in nutrition behaviours: Targeted interventions needed. J Nutr Educ Behaviour. 2010; 42(1):10-6. doi: 10.1016/j.jneb.2008.11.003.

34. Kpodo FM, Mensah C, Dzah CS. Fruit and vegetable consumption patterns and preferences of students in a Ghanaian Polytechnic. World Journal of Nutrition and Health. 2015; 3(3): 53-59. Doi: 10.12691/jnh-3-3-2.
35. Layade AA, Adeoye IB. Fruit and vegetable consumption among students of tertiary institutions in Oyo State. RJOAS. 2014; 6(30): 1-6.
36. Doku DT, Koivusilta L, Raisamo S, Rimpela A. Socio-economic differences in adolescents' breakfast eating, fruit and vegetable consumption and physical activity in Ghana. Public Health Nutrition. 2011; 27, 1-9.
37. Othman KI, Abdul KSR, Karim R, Adzhan N, Abdul HN, Osman S. Factors influencing fruits and vegetables consumption behaviour among adults in Malaysia. Journal of Agribusiness Marketing. 2012; 5, 29- 46.
38. Currie, C., Nic Gabhainn, S., Godeau, E., Roberts, C., Smith, R., Currie, D., Pickett, W., Richter, M., Morgan, A., & Barnekow, V. (eds.). Inequalities in young people’s health: HBSC international report from the 2005/2006 survey, health policy for children *and adolescents number* 5. Copenhagen: WHO Regional Office for Europe. 2008.
